# The financial burden of complications following rectal resection: A cohort study

**DOI:** 10.1097/MD.0000000000020089

**Published:** 2020-05-08

**Authors:** Samuel A. Johnston, Maleck Louis, Leonid Churilov, Ronald Ma, Nada Marhoon, Andrew Bui, Christopher Christophi, Laurence Weinberg

**Affiliations:** a University of Melbourne, Parkville; b Department of Anesthesia; c Department of Medicine, Austin Health, Heidelberg; d Melbourne Brain Centre, Royal Melbourne Hospital, Parkville; e Department of Finance; f Data Analytics and Research Centre; g Department of Surgery; h Department of Surgery, University of Melbourne, Parkville, Victoria, Australia.

**Keywords:** complications, cost, rectal, surgery

## Abstract

To investigate the costs associated with postoperative complications following rectal resection.

Rectal resection is a major surgical procedure that carries a significant risk of complications. The occurrence of complications following surgery has both health and financial consequences. There are very few studies that examine the incidence and severity of complications and their financial implications following rectal resection.

We identified 381 consecutive patients who underwent a rectal resection within a major university hospital. Patients were included using the International Classification of Diseases (ICD) codes. Complications in the postoperative period were reported using the validated Clavien-Dindo classification system. Both the number and severity of complications were recorded. Activity-based costing methodology was used to report financial outcomes. Preoperative results were also recorded and assessed.

A 76.9% [95% CI: 68.3:86.2] of patients experienced one or more complications. Patients who had a complication had a median total cost of $22,567 [IQR 16,607:33,641]. Patients who did not have a complication had a median total cost of $15,882 [IQR 12,971:19,861]. The adjusted additional median cost for patients who had a complication was $5308 [95% CI: 2938:7678] (*P* < .001). Patients who experienced a complication tended to undergo an open procedure (*P* = .001), were emergent patients (*P* = .003), preoperatively had lower albumin levels (36 vs 38, *P* = .0003) and were anemic (*P* = .001).

Complications following rectal resection are common and are associated with increased costs. Our study highlights the importance of evaluating and preventing complications in the postoperative period.

## 1 Introduction

Colorectal cancer is the third most common malignancy and the fourth leading cause of cancer-related death worldwide.^[1]^ It is predicted that colorectal cancer around the globe will increase by 60%, leading to 2.2 million new cases and 1.1 million deaths by 2030.^[2]^ Within Australia, the incidence of rectal cancer is increasing amongst those who are aged 50 years or less.^[3]^ Current mainstay strategies for treating rectal cancer involve surgery, with an uptake in the use of laparoscopic surgery from 2.4% in 2000 to 27.5% in 2007 to 2008 within Australia.^[4]^

There is considerable variation in the incidence and types of complications following rectal resection.^[5]^ The significance of complications occurring following rectal resection have clinical consequences. These include increased length of stays, poorer oncological outcomes and a negative association with 5-year disease-free survival after surgery.^[6]^ In combination with clinical consequences, there is an association of increased costs for the occurrence of complications.^[7]^ This has future implications on the provision of healthcare, both in terms of the quality and quantity that can be delivered. Additionally, there is evidence to suggest that preoperative factors, including anemia and hypoalbuminemia, are associated with increased costs following surgery.^[8,9]^

However, there are very few studies that examine the incidence and severity of complications and their financial implications following rectal resection. To address this, we conducted a study examining the proportion and severity of complications following rectal resection. Our primary aim was to estimate the cost differences between patients with and without complications. Our secondary aims included estimating the amount and severity of complications whilst also examining preoperative patient characteristics and operative technique. We hypothesised that postoperative costs would increase when complications occurred, and that there will be a dose-response relationship between the costs and the amount of complications that occurred and their severity.

## 2 Methods

After Human Research Ethics Committee approval (Number: LNR/18/Austin/358) and registration with the Australian and New Zealand Clinical Trials Registry (ANZCTR) (Registration Number: ACTRN12619000806167), we performed an observational cohort study at a university teaching hospital with a high-volume workload for colorectal surgeries. Inclusion criteria were adult patients (≥18 years old) undergoing emergent or elective rectal resection surgery between January 1, 2013 and June 30, 2018. Data was sourced from the Data Analytics Research and Evaluation (DARE) Centre, the Department of Finance and the electronic health record system *Cerner*, which are all based at Austin Health.

### 2.1 Definitions

The following ICD codes were utilized in identifying eligible patients for inclusion: “Ultra low anterior resection of rectum,” “High anterior resection of rectum,” “Rectosigmoidectomy with formation of stoma,” “Ultra low anterior resection of rectum with hand sutured coloanal anastomosis,” “Laparoscopic rectosigmoidectomy with formation of stoma,” “Total proctocolectomy with ileo-anal anastomosis,” “Abdominoperineal proctectomy,” “Low anterior resection of rectum,” “Anterior resection of rectum, level unspecified,” “Total proctocolectomy with ileostomy,” “Total proctocolectomy with ileo-anal anastomosis and formation of temporary ileostomy,” “Perineal rectosigmoidectomy,” “Perineal proctectomy.” Patients who had a concomitant procedure were also included.

Complications were defined as any deviation from the normal course in the postoperative period. The classification of complications was guided by the European Perioperative Clinical Outcome (EPCO) definitions,^[10]^ the Classification of Hospital Acquired Diagnoses (CHADx)^[11]^ and reported clinical information from the hospital's electronic health record system. Complication severity was graded utilizing the Clavien-Dindo classification system,^[12]^ which is a validated method to compare different complications and their severity. Two authors (SJ and ML) were involved in the coding and classification of complications. Where there was a discrepancy regarding the classification of a complication, this was resolved by consensus from a third author (LW). Length of stay was calculated from the date of the operation until the day of discharge and excluded Hospital in the Home (HITH) and “patient leave” days. Readmission was defined as an unplanned hospital admission within 30 days of the date of discharge. Mortality was considered within the index admission episode. Anemia was defined in accordance with standardized pathology measures as a value <120 g/L for females and a value <130 g/L for males.

### 2.2 Study outcomes

Our primary outcome was to estimate the additional cost associated with postoperative complications following rectal resection.

Our secondary outcome was to report the preoperative patient characteristics associated with the presence of complications, and to examine the additional cost associated with the severity and number of complications that occurred amongst patients.

### 2.3 Cost analysis

Costs were calculated from the day of the qualifying operation until the day of discharge. This was performed using activity-based costing methodology. All costs incurred during this period were included and only inpatient costs were considered. If a patient had an unplanned readmission, the cost of that readmission was also included. Itemized costs were analysed and grouped together based on common service areas. These cost groupings were then summed together to derive total cost.

Inflation was accounted for by using the Consumer Price Index (CPI) provided by the Australian Taxation Office (ATO).^[13]^ Based on the operation date, the corresponding CPI for that financial quarter was applied and the costs adjusted accordingly. Costs were converted from Australian Dollars ($AUD) to United States Dollars ($USD) based on the market rate on the March 31, 2019. Costing results were expressed to the nearest whole $USD.

### 2.4 Statistical analysis

The raw data was assessed to see if missing data impacted significantly on the statistical analysis. Appropriate statistical adjustment was used according to the degree and type of missing data (if necessary). To investigate the costs associated with complications following rectal resection, we used bootstrapped quantile regression with the presence, amount or severity of complications as the independent variable, total cost as the dependent variable, and surgical urgency, operative technique, Charlson Comorbidity Index (CCI) and preoperative anemia status a-priori selected covariates. Quantile regression models the association between a set of input variables and specific percentiles (or quantiles) of the outcome variable and estimates differences in the quantiles of the outcome variable between total cost and the presence, amount or severity of complications. For each outcome, we included three quantile regression models: the 25th percentile, the 50th percentile (median), and the 75th percentile. All calculated *P*-values were two-sided. A *P*-value of ≤.05 was considered significant. Violin plots of the unadjusted total costs were also provided to compare the calculated adjusted quantile regression values, and to visualize the distribution of the costing data. This was performed for both the number and severity of complications.

To investigate the association of patient associated factors and complications, we used either Wilcoxon–Mann–Whitney test or Kruskal–Wallis test for continuous variables and the Fisher's exact test or Poisson exact test for categorical variables. Due to the multiple comparisons being made, we used the two-sided Bonferroni-corrected threshold (*P* = .003125) for identifying statistical significance.

Statistical analysis was performed utilizing STATA/IC v.15.1.

### 2.5 Data integrity

An audit utilizing a random number generator was undertaken by two authors (SJ and ML) to confirm the accuracy of the entered data. If the sampled data contained errors of more than 10% of the corresponding variable, the variable would be fully reviewed and cross-checked by two authors (SJ and ML).

## 3 Results

### 3.1 Patient characteristics

We identified 381 patients who underwent a rectal resection at Austin Health between January 2013 and July 2018. The median age was 64 years [IQR 54:73] with there being 220 (57.7%) males and 161 (42.3%) females. The median body mass index (BMI) was 27.03 kg/m^2^ [IQR 23.39:30.81] with 75 (21.6%) patients identified as having been smokers within 1 year of their operation. 9 (2.7%) patients were identified as consuming >5 standard drinks of alcohol per day. The median CCI amongst patients was 6 [IQR 4:8]. Two hundred fifty-nine (68.0%) patients underwent a resection for a malignant condition while 122 (32.0%) patients underwent a resection for a benign condition. Sixty-three (16.5%) patients received chemotherapy within 3 months of their operation.

There were 93 (24.4%) emergent cases and 288 (75.6%) non-emergent cases. A total of 329 (86.3%) patients were public patients with 48 (12.6%) being private patients. Thirty-two (9.6%) patients had a previous bowel resection. Laparoscopy was the most commonly used surgical technique which was used with 238 (62.5%) patients. There were 143 (37.5%) patients who had an open technique used. Patients who had a laparoscopic procedure that was converted to an open procedure were included in the open group. Patients who had a laparoscopic-assisted procedure were included in the laparoscopic group. There were 37 (9.7%) patients whose laparoscopic procedure was converted to an open procedure. There were 29 (7.6%) laparoscopic-assisted procedures. Of the conducted procedures, 59 (15.5%) were identified as being concomitant with another unrelated procedure.

### 3.2 Cost analysis

Patients who had at least one complication had a median total cost of $22,567 [IQR 16,607:33,641]. In comparison, patients who did not have a complication had a median total cost of $15,882 [IQR 12,971:19,861]. The adjusted additional median cost for patients who had a complication was $5308 [95% CI: 2938:7678] (*P* < .001). Patients who experienced just one complication had an adjusted additional median cost of $2694 [95% CI: 337:5051] (*P* = .025) as compared to those without any complications. For each additional complication a patient had, this was associated with an increased adjusted median additional cost (for every category *P* < .05). Patients having four or more complications had the highest adjusted median additional cost of $21,105 [95% CI: 16,430:25,780] (*P* < .001). There was a dose–response relationship between the adjusted median additional cost and complication severity, except for a Grade I and Grade V complication. A Grade I complication had an associated reduced adjusted median cost of −$7 [95% CI: −1905:1890] (*P* = .994) in comparison with patients who had no complications. A Grade V complication had an associated adjusted additional median cost of $13,449 [95% CI: −3080:29,977] (*P* = .110). The most severe complication without mortality (Grade IV) had an adjusted additional median cost of $25,998 [95% CI: 16,770:35,227] (*P* < .001) (see Table 1).

**Table 1:**
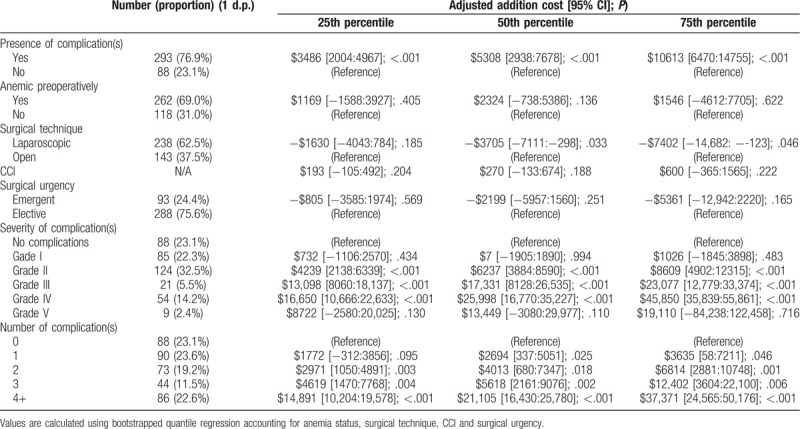
Adjusted additional cost amongst patients with or without complications.

**Figure 1:**
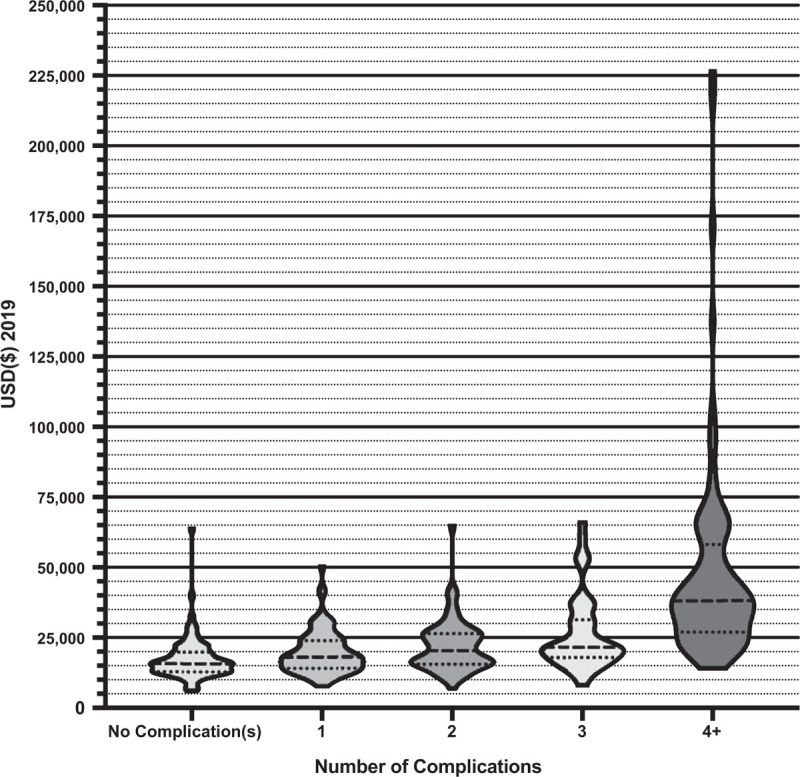
Violin plots with quartiles of the unadjusted total costs associated with the number of complications experienced by patients.

**Figure 2:**
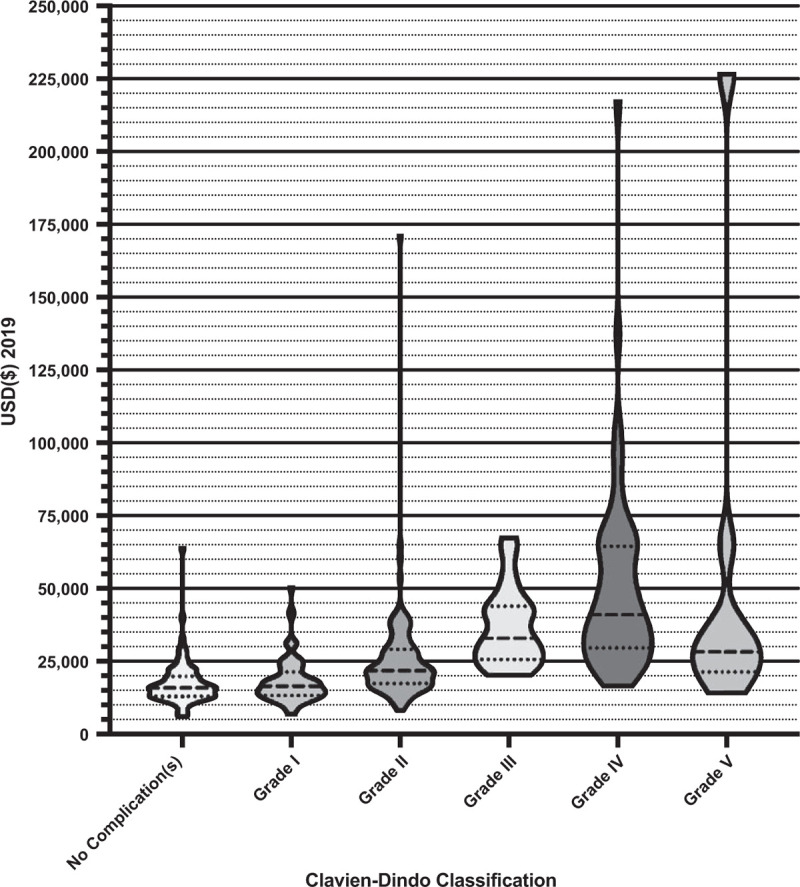
Violin plots with quartiles of the unadjusted total costs associated with the severity of complications experienced by patients.

There was a significant range and distribution of unadjusted total costs associated with the number and severity of complications (see Figs. 1 and 2). For unadjusted total cost, the highest total cost of an admission was $226,694 for a patient who had 11 complications and experienced a Grade V as their most severe complication. The median cost of a readmission was $4062 [IQR 2172:9173]. Some of the largest contributors to total expenditure for all patients were operative (30.7%), ward (24.3%) and intensive care unit (ICU) (10.3%) costs.

### 3.3 Complications

A total of 293 (76.9% [95% CI: 68.3:86.2]) patients experienced one or more complications during their postoperative period. The demographic, clinical and process of care characteristics of patients with and without complications are presented in Table 2. Those patients who experienced a complication were emergent patients (*P* = .003), preoperatively had lower albumin levels (36 vs 38, *P* = .0003) and were anemic (*P* = .001), and tended to undergo an open procedure (*P* = .001). Mortality amongst the cohort was 2.4% (n = 9). The median amount of complications that occurred for patients who had a complication was 2 [IQR 1:4]. The maximum number of complications experienced by a patient was 12.

**Table 2:**
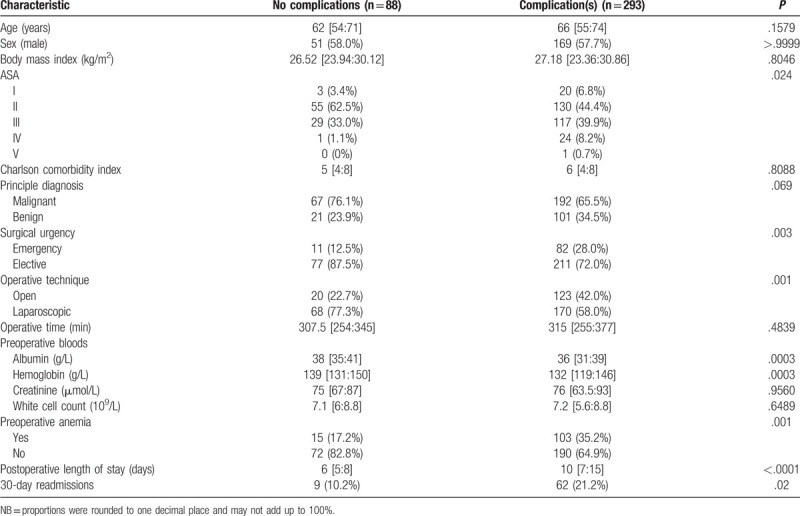
Patient characteristics by presence of complications, presented as the median (IQR) and number (proportion).

If complications were dichotomized into minor (Grade I–II) and major (Grade III–V) complications, of patients who had a complication, 209 (71.3%) patients had a minor complication. Only 84 (28.7%) patients experienced a major complication.

Patients who had a complication had an increased median length of stay by 4 days (*P* < .001) as compared to those patients who did not have a complication. Aside from the stated differences, the cohorts were largely similar. The proportion of patients who had a readmission was higher in patients who had a complication (21.2% vs 10.2%; *P* = .02). The median amount of days after discharge for an unplanned readmission to occur amongst patients was 7 [IQR 3:11]. Seventy-two (18.9%) patients received a red blood cell transfusion. For those that had received a red blood cell transfusion, the median number of transfused units was 2 [IQR 1:4].

## 4 Discussion

We conducted a detailed analysis on the burden of complications on cost for all patients who underwent a rectal resection at a high-volume university hospital. Our findings were in line with our hypotheses in that patients who experienced a complication had a greater additional median cost than those that did not have a complication and that this had a dose-response relationship to the amount of complications a patient had. For severity, there was an associated dose–response relationship, except for Grade I and Grade V complications. Complications amongst this cohort were common, with major complications (Grade III–V) being experienced by around one in five patients. The majority of complications were minor complications (Grade I–II). Patients who experienced a complication were an emergent case, preoperatively anemic, had lower preoperative albumin levels, and tended to undergo an open procedure

It is difficult to assess whether our overall complication incidence of 76.9% [95% CI: 68.3:86.2] is higher or lower than expected. There is considerable heterogeneity amongst studies that reported on the complication incidence amongst patients who underwent a rectal resection.^[14–18]^ Most of these studies either did not use the Clavien-Dindo system of classification,^[12]^ only reported on a set of certain complications or neglected to account for minor complications. Given this, we believe our complication rate is high due to our study being comprehensive in including all minor and major complications. Our comparison between the incidence of complications for an open versus laparoscopic approach was also higher than reported literature.^[5,19–21]^ We believe this is also due to these studies neglecting to account for minor complications.

By using activity-based costing methodology, we were able to show that the impact of complications on cost was significant in both amount and severity. Patients who just experienced one complication during their admission tended to cost over $2000 more than patients who did not have a complication. In terms of severity, there was not a significant associated increase in the adjusted median additional cost for patients who just experienced a Grade I complication. A Grade I complication under the Clavien-Dindo^[12]^ classification system is a complication that can only be treated with agents such as antipyretics, electrolytes, diuretics or with bedside wound care. It could be that due to the minimal amount of acceptable interventions allowed for treating Grade I complications, their associated costs are minimal as compared to patients with no complications. There was an association of an increased adjusted median additional cost for patients who experienced a Grade V complication. However, this was statistically insignificant. A Grade V complication is a complication that involves mortality. Often patients who suffer a Grade V complication have complicated medical and surgical histories, placing them at an increased risk of major complications. We attribute the statistically insignificant associated increased adjusted median cost due to the small population of patients experiencing a Grade V complication (n = 9). Just experiencing a minor complication (Grade II) tended to approximately cost $6000 more in comparison to those patients who did not have a complication. Common examples of minor complications in our study included hypovolemia and nutritional deficiency requiring total parenteral nutrition (TPN). We found that over 70% of complications experienced by patients were minor complications. In our study, we have demonstrated the significance of minor complications (Grade II) on cost outcomes. Under the Clavien-Dindo classification system,^[12]^ acceptable means of treating complications that are classified as a Grade II usually involve pharmacotherapy or basic first-aid. With growing attention around unnecessary resource consumption within hospitals, and that minor complications were the most common form of complication, this represents a significant and feasible area for hospitals to target in order to reduce their costs. We believe that by reporting on minor complications, we have added important information to the existing literature and have been able to identify an area for hospitals to target to reduce costs that may otherwise have been neglected.

In contrast, patients who experienced a major complication without death (Grade III–IV) tended to have adjusted additional median costs around two and half times greater compared with patients who had minor complications. Treating complications that are either a Grade III or Grade IV involve surgical/radiological intervention and ICU support respectively. Not only does this represent a significant negative effect on a patient's potential healthcare outcomes, it also represents a significant cost burden to hospitals. We recommend that hospitals continue to place importance on reducing the occurrence of complications, in particular major complications, from both a clinical and cost outcome point of view.

The additional cost incurred by patients who had complications can largely be attributed to the additional resources required to treat the complications, including additional medical consultations, increased medications, ICU admissions, reoperations and readmissions. Importantly, patients who had complications had longer associated median length of stays. Not only do increased length of stays represent a greater cost for hospitals, they also prevent the admission of new patients for treatment, particularly when hospitals are at capacity. This puts further pressure on hospitals and may lead to a compromise in care given. In addition to this, the presence of complications was also an indicator of whether a patient would have an unplanned readmission within 30 days of discharge. Readmissions also place increased pressure on a hospital's service capacity.

There are several strengths to our study. We have provided a comprehensive analysis regarding the impact of complications on cost following rectal resection. We have achieved this by using the Clavien-Dindo classification,^[12]^ which provides a standardized method of comparing complication severity. Furthermore, we have analysed the relationship of cost against the number and severity of complications that occur. Our study also has focussed on all complications, regardless of type and severity. We are also one of the first studies to use regression analysis accounting for anemia status, surgical technique, CCI and surgical urgency on the associated additional median cost of complications. This also included report on the amount and severity of complications that occurred.

There are limitations to our study. Our study focuses on patients who underwent rectal surgery. This was undertaken as we hypothesized that the costing data would be different across different surgical patient sub-groups. By utilizing specific surgical candidate criteria, further studies in this area can be more accurately compared in future systematic reviews. From this approach, we were able to model our data with a higher degree of specificity in regard to patients who underwent rectal surgery. We also completed this study retrospectively and so there may be a degree of selection and information bias. However, this was minimized by using two authors (SJ and ML) for classifying complications, utilizing electronic health records and by having cost and patient data provided by external departments, which collect data independently. We further reduced misinformation bias by performing a random audit of the database to ensure accuracy. The retrospective nature of this study meant that there was some missing data. However, we identified that the degree of missing data was at an acceptable level and would not greatly impact this study's outcomes. The authors encourage the use of methods that enable prospective studies to be performed with regards to tracking costing data. Our study was also completed within a single university hospital and may not be representative of the broader population. Our study only takes into account cost and we were not able to provide an economic-adjusted analysis on complications and their cost and healthcare outcomes. This includes a quality of life and cost-effectiveness analysis following rectal resection.

## 5 Conclusion

Complications following rectal resection are common and are associated with increased costs. The impact of complications on cost has a dose-response relationship to both the amount and severity of complications, except in the case of Grade I complications and mortality (Grade V). Our study highlights the importance of evaluating and preventing complications in the postoperative period. It allows health institutions to review their practices in addressing complications and their associated cost, and encourages further studies to expand on potential identification and mitigation strategies to address complications and cost into the future.

## Author contributions

Samuel A Johnston: study design, data collection, manuscript writing and editing; leck Louis: data collection and manuscript editing; Leonid Churliov: statistical analysis and manuscript editing; Ronald Ma: financial analysis and manuscript editing; Nada Marhoon: data collection and manuscript editing; Andrew Bui: study design and manuscript editing; Christopher Christophi: study design and manuscript editing; Laurence Weinberg: study conception and design, data analysis, manuscript writing and editing.
